# 3.2 PARVALBUMIN INTERNEURON IMPAIRMENT INDUCED BY OXIDATIVE STRESS AS A COMMON PATHOLOGICAL MECHANISM IN ANIMAL MODELS OF SCHIZOPHRENIA

**DOI:** 10.1093/schbul/sby014.004

**Published:** 2018-04-01

**Authors:** Jan Harry Cabungcal, Pascal Steullet, Joseph Coyle, Michael Didriksen, Kathryn Gill, Anthony Grace, Hensch Takao, Anthony LaMantia, Lothar Lindemann, Thomas Maynard, Urs Meyer, Hirofumi Morishita, Patricio O’Donnell, Matthew Puhl, Michel Cuenod, Kim Q Do

**Affiliations:** 1 Center for Psychiatric Neuroscience, Lausanne University Hospital; 2 Laboratory for Psychiatric and Molecular Neuroscience, Harvard Medical School, McLean Hospital; 3 H. Lundbeck A/S; 4 University of Pittsburgh; 5 Harvard University; 6 George Washington Institute for Neuroscience, The George Washington University; 7 F. Hoffmann-La Roche Ltd., Roche Pharmaceutical and Early Development, Neuroscience, Ophthalmology & Rare Disease (NORD) DTA, Discovery Neuroscience, Roche Innovation Center Basel; 8 Institute of Pharmacology and Toxicology, University of Zurich-Vetsuisse; 9 Friedman Brain Institute, Mindich Child Health and Development Institute, Icahn School of Medicine at Mount Sinai; 10 Pfizer, Inc

## Abstract

**Background:**

Parvalbumin inhibitory interneurons (PVIs) are crucial for maintaining proper excitatory/inhibitory balance and high-frequency neuronal synchronization. Their activity supports critical developmental trajectories, sensory and cognitive processing, and social behavior. Despite heterogeneity in the etiology across schizophrenia and autism spectrum disorder, PVI circuits are altered in these psychiatric disorders. Identifying mechanism(s) underlying PVI deficits is essential to establish treatments targeting in particular cognition. Based on our previous publications and new data, we propose oxidative stress as a common pathological mechanism leading to PVI impairment in schizophrenia and some forms of autism.

**Methods:**

Using immunohistochemistry technique and confocal imaging analysis, we assessed the relationship between oxidative stress (as revealed by 8-oxo-DG immunolabeling) and PVI and their perineuronal net (PNN) in twelve established animal models relevant to autism (i.e., the fmr1 KO and CNV 15q13.3) and schizophrenia (CNV: 22q11, 15q13.3, 1q21, serine racemase (SR) KO, GRIN2A KO, Gclm KO) with or without additional insult (e.g., environmental: Gclm KO + GBR12909, GRIN2A KO + GBR12909, neonatal ventral hippocampal lesion (NVHL), methylazoxymethanol acetate developmental rodent model (MAM) and poly:IC).

**Results:**

When PVI deficits in the anterior cingulate cortex were found in these animal models carrying genetic and/or environmental risks relevant to diverse etiological aspects of these disorders, oxidative stress was always present. Specifically, oxidative stress was negatively correlated with the integrity of PVIs and the extracellular perineuronal net enwrapping these interneurons. Oxidative stress may result from dysregulation of systems typically affected in schizophrenia, including glutamatergic, dopaminergic, immune, and antioxidant signaling. As convergent endpoint, redox dysregulation has successfully been targeted to protect PVIs with antioxidants/redox regulators across several animal models (e.g., Gclm KO, NVHL rats, GRIN2A KO and SR KO mice). D-serine, an allosteric modulator of brain NMDA receptor also protected PVIs and PNN against oxidative stress in SR KO mice.

**Discussion:**

In view of the fact that the established pathophysiological processes dopamine excess, immune dysregulation and NMDA receptor hypofunction could all induce oxidative stress and are potentiated by additional oxidative insults, this mechanism could be central to damage of the highly metabolically active PVIs and the PNN surrounding them. Antioxidant systems are therefore potential therapeutic targets, assuming that redox regulators could be applied early, during environmental impacts, long before the clinical emergence of the disease.

